# Recent Advances in the Development of Nano-Sculpted Films by Magnetron Sputtering for Energy-Related Applications

**DOI:** 10.3390/nano10102039

**Published:** 2020-10-15

**Authors:** Adriano Panepinto, Rony Snyders

**Affiliations:** 1 Chemistry of Plasma-Surface Interactions, University of Mons, 23 Place du Parc, 7000 Mons, Belgium; rony.snyders@umons.ac.be; 2 Materia Nova Research Center, Chemistry of Plasma-Surface Interactions, 3 Avenue Nicolas Copernic, 7000 Mons, Belgium

**Keywords:** magnetron sputtering, GLAD, nano-sculpted films, growth simulations, DSSCs

## Abstract

In this paper, we overview the recent progress we made in the magnetron sputtering-based developments of nano-sculpted thin films intended for energy-related applications such as energy conversion. This paper summarizes our recent experimental work often supported by simulation and theoretical results. Specifically, the development of a new generation of nano-sculpted photo-anodes based on TiO_2_ for application in dye-sensitized solar cells is discussed.

## 1. Introduction

In addition to more conventional features such as the thickness, chemical composition, or phase constitution, the control of the morphology at the submicrometer scale and the associated porosity is accepted as an essential parameter, allowing the properties of thin films to be tailored. Yet, for many years, the design and fabrication of nano-sculpted thin films have been recognized as a new opportunity to improve the performance of the thin films in a wide variety of applications ubiquitous in our society in the fields of microelectronics, information processing, as well as energy generation and storage. [1] The main interest toward nano-sculpted films mainly arises from their high surface-to-volume ratio, allowing for an important developed surface that can be used as examples to accommodate a large quantity of molecules (i.e., in dye-sensitized solar cells) or to strongly increase the active sites (i.e., in photocatalytic materials).

Specifically addressing the domain of solar energy harvesting, one of the most frequent nano-sculpted film architectures is based on fritted nanoparticles thin films [2]. Such a structure, which is the one used nowadays in dye-sensitized solar cells (DSSCs) as an example, often presents a very high specific surface area and very good porosity, which is crucial to improving the dye absorption in the mentioned application. Nevertheless, they also present a dramatic limit in terms of the relatively low quality of charge carriers transport [3,4]. This problem is often associated with the scattering of the charge carriers at the numerous grain boundaries between the fritted particles constituting the film [5]. This has motivated the development of thin films based on one-dimensional (1D) nanostructures, expected to present a much better facility to transport charge through the film. In reality, such a thin film consists of a tri-dimensional (3D) arrangement constituted of nano-objects grown with a preferential direction from the substrate. This means that two of their dimensions are less than 100 nm while the other can reach several micrometers [6]. Individually, such a 1D object would avoid the carriers’ transport limitations by creating direct pathways through the material without particle interconnections, while keeping a high specific surface area. As a consequence, the transport path for the charge collection is efficiently shortened and, in most cases, these materials have higher electron diffusion coefficients than nonordered nanostructures [7], allowing a large increase in the thickness of the film. The price to pay for such an architecture is an often lower specific surface area, as well as porosity, in comparison with the conventional nanoparticles-based thin-film architecture. Indeed, for the latter, the conventional specific surface area and porosity are ~200 m^2^/g and >75%, respectively, while the best reported values for 1D nanostructures-based films are ~140 m^2^/g and 70%, respectively [8,9]. This has motivated many efforts to improve such a structure in the last few years. Basically, researchers have been focused on the improvement in the design of these hierarchical nanostructures, aiming to reach an optimal equilibrium between the specific surface area, porosity, and charge transport efficiency. As a noteworthy example, Kuang et al. established a systematical strategy to grow TiO_2_ hierarchical nanostructures made of nanowires (NW) on which nanorods (NR) are branched, themselves being sources of nanorods (NR), labeled as NW/NR/NR. These hierarchical structures have been utilized in solar cell applications, allowing for a conversion efficiency of up to 9% due to a larger specific surface area, lower transport time, and longer electron lifetime than TiO_2_ nanoparticles [10]. From these results, they concluded that the design of 1D hierarchical nanostructure thin films is one of the keys to find the best agreement between a high carriers’ reservoir capability and good charge transport properties in DSSCs. 

Numerous processes have been used to synthesize such nano-sculpted materials: Anodic oxidation [11], electron beam evaporation [12], atomic layer deposition [13,14], sol–gel deposition in a template [15,16], or hydrothermal methods, which has been intensively utilized by successive treatments to obtain branched nanowires [17,18]. However, these methods are usually difficult to industrialize, often lead to the synthesis of amorphous materials, and make necessary the use of solvents and toxic chemicals. In this context, it is necessary to develop industrially viable alternative synthesis roads for such structures that would allow a crystallized material to be synthesized with a low environmental impact. Physical vapor deposition (PVD) techniques are well-established in various manufacturing areas such as microelectronics, automotive, and biomedical industries [19]. In these fields, plasma-assisted processes are generally preferred to thermal evaporation in response to requirements of materials processing at reasonable temperatures. In PVD processes, the target material to be deposited as a thin film is transformed into the vapor phase by different means, generally involving plasma generation (except for thermal evaporation). The chemical composition of the deposited film can be tuned by the addition of various reactive gases (O_2_, N_2_, etc.) in order to form oxides, nitrides, or more complex compounds, which makes the technique versatile.

As a widespread plasma technology, magnetron sputtering, which belongs to the PVD methods and consists of bombarding a target material with accelerated ions from the plasma, leading to the ejection of particles (mainly atoms and clusters), has been used to build the thin film. Oxides, nitrides, or carbides can also be grown by adding pure O_2_, N_2_, or C-based vapor sources inside the magnetron sputtering deposition chamber, the so-called reactive magnetron sputtering (RMS) regime [20,21]. The magnetron sputtering process offers the opportunity for tuning the crystalline constitution depending on the energy brought to the growing film by adjusting the experimental parameters such as the applied power [22]. In most of the aforementioned applications, magnetron-deposited films are meaningful because they are dense, homogeneous, and chemically pure [23]. 

**Figure 1:**
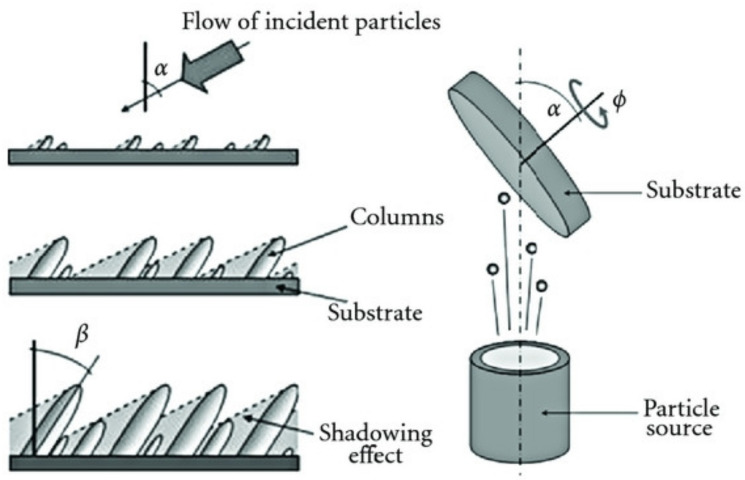
Schematic description of the ballistic shadowing effect during thin-film growth in glancing angle geometry (reproduced from [25], Hindawi, 2012).

In this work, in order to generate a 1D structure-based thin film, the so-called nano-sculpted films, we have utilized magnetron sputtering in glancing angle geometry. Glancing angle deposition (GLAD) is a particular case of oblique deposition where the substrate position is manipulated during the film deposition [24]. The technique takes advantage of the ballistic shadowing effect, which allows the formation of columnar microstructures as the film is growing. The basic operation principle is presented in Figure 1 and can be summarized as follows: While the substrate is tilted with an angle α compared to the target normal, the initial nuclei of the depositing film randomly roughen the surface. Subsequently, the depositing particles nucleate on the substrate, while the region behind the nucleus does not receive any vapor, because it falls in the shadow of the nucleus. Consequently, a larger number of particles will be deposited onto the nuclei than in the shadowed area. This inequality increases as growth continues. As only the tops of the nuclei receive the depositing material, the nuclei will develop into columns, tilted in the direction of the incident particles flux and forming an angle *β* with the substrate normal (*β* < *α*). The *β* value depends on many experimental parameters as it will be discussed in this paper.

The key principle of GLAD consists of changing the vapor flux direction to operate ballistic shadowing and to provide control over the final thin-film morphology. Two degrees of freedom are obtained by tilting the substrate with respect to the source of particles (*α*), while rotating the substrate around its normal axis allows the substrate azimuthal angle *ф* to be controlled with a fixed substrate rotation speed (*ф*_s_). Varying *ф* modifies the direction of the incident vapor flux and provides control over the shadowed regions of the substrate.

There are mainly four archetypal columnar microstructures, which illustrate how substrate motion affects the microstructure. Basically, inclined columns are obtained when working with a fixed tilt angle (*α*) higher than 60°; zig-zags are generated when rotating the substrate by successive rotation of the substrate by a 180° angle in the latter configuration; and plots or helical structures can be grown continuously, rotating the tilted substrate during deposition [9]. All of these structures are generated by modifying the substrate rotation as *α* is unchanged for each one. The ability to sculpt the film and access the various morphologies is provided by the trajectory of the incident vapor flux relative to the substrate surface during deposition. Accordingly, each nanostructure is characterized by a given porosity (inter-columnar space) that mainly depends on the columnar tilt.

It is important that the incident vapor remains highly directional to avoid merging of the shadowed regions. Indeed, deposition with a poorly collimated incident flux allows vapor to directly access the shadowed area. This implies that the mean free path of the particles should be greater than the distance to the substrate. It is thus evident that PVD techniques such as thermal and electron beam evaporation are the most prevalent in GLAD research because they allow high target-to-substrate distances and low operating pressures with small vapor sources. Therefore, GLAD has mainly been utilized in combination with an evaporation source to grow various nano-sculpted materials [26]. Nevertheless, using evaporation, the energy brought to the growing film does not allow us to control the crystalline structure of the deposited films in most of the cases. By contrast, as mentioned, magnetron sputtering is a recognizable technique to do this even without intentional heating of the growing material. Indeed, in this case, the crystallization is promoted by the bombardment of energetic particles, as well as infrared radiation emitted from the target during the sputtering process [27]. Although promising, the utilization of GLAD geometry in combination with magnetron sputtering, i.e. magnetron sputtering in glancing angle geometry (MS-GLAD), is surprisingly quite recent [28], and has attracted considerable interests for 10 years [29].

In this paper, we aim to overview the work that has been developed in our group during the past few years, utilizing this original synthesis process to design nano-sculpted thin films that are ultimately utilized in energy-related applications. Due to the unusual character of the MS-GLAD, we first had to answer many questions related to the growth mechanism of the nano-sculpted films by this approach in order to be able to control the synthesized films features (morphology, chemistry, crystalline constitution, etc.). Indeed, if magnetron-sputtered thin films can be crystallized by a higher supply of energy, the price to pay is the generation of dense films, inhibiting the effect of the grazing mode configuration [30]. In order to obtain a full picture of the growth mechanism, experimental as well as simulation works have been developed. Finally, we will summarize the benefits that can be associated with the utilization of these nano-sculpted films in energy-related applications, specifically as the photo-anode in dye-sensitized solar cells (DSSCs), although other applications of our films have been investigated in our group [31].

## 2. Materials and Methods

### 2.1. Experimental Setup

**Figure 2:**
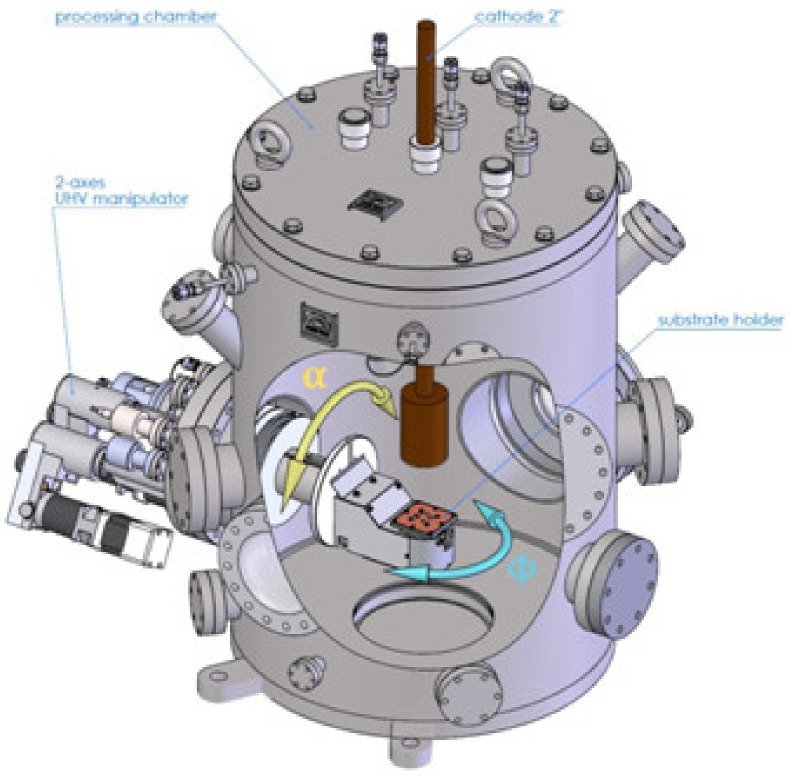
Sketch of the deposition chamber used in this work (reproduced from [32], MDPI, 2019).

The nano-sculpted films were synthesized in a cylindrical stainless-steel magnetron sputtering chamber (height: 60 cm, diameter: 42 cm), schematically presented in Figure 2. The chamber was evacuated down to a residual pressure of 10^−4^ Pa by a turbo molecular pump (Edwards nEXT400D 160W, Edwards, Irvine, California, CA, USA), backed by a dry primary pump (Edwards nXDS10i, Edwards, Irvine, California, CA, USA).

An unbalanced magnetron cathode was installed in front of the substrate, at the top of the chamber on which a 2 in. (5.08 cm) diameter and 0.25 in. (0.635 cm) thick target was connected. The target/substrate distance was fixed at 7 cm. Pure Ti and Mg (both with 99.99% purity) were used as target materials in this study. In order to modify the phase constitution of the deposited films, the target was sputtered either in direct current (DC) mode or in the high power impulse magnetron sputtering (HiPIMS) regime. For the DC mode, an Advanced Energy MDK 1.5 K (Advanced Energy, Denver, Colorado, CO, USA) power supply was used. The power (P) was fixed at 150 W, corresponding to a power density on the target surface of 7.5 W·cm^−2^, which was calculated by taking into account the target surface exposed to the plasma (~20 cm^2^). In the HiPIMS regime, a lab-made power supply based on a one-quadrant chopper topology was used, allowing the generation of short high-power pulses at the cathode [33]. We have to mention that the discharge voltage is measured at the output of the lab-made power supply, not directly at the cathode. More information can be found elsewhere [34].

Argon, which is the sputtering gas with or without O_2_ as the reactive gas (both with 99.999% purity), was introduced in the chamber using two distinct mass flow meters in order to grow oxide or metallic compound, respectively. Note that the gases were mixed prior to being injected in the vacuum chamber. All thin films were deposited at constant total gas flux. It was fixed at 15 sccm (standard cubic centimeter per minute) to allow low working pressure (0.13 Pa) according to the pumping rate.

The substrate was installed on a 2-axis manipulator, allowing two rotation motions: Along the *α* angle to tilt the substrate from *α* = 0° to *α* = 90° with regard to the cathode axis and/or along the *ф* angle to rotate the substrate step by step or in continuous mode with a given angular speed *ф*_s_. The α and *ф* angles were varied in order to generate various morphologies: Discrete rotations (*ф* = +180° or −180°) allow zigzag structures to be grown, while continuous rotations (*ф*_s_ = 0.1, 1.0 or 10°/s) lead to vertical pillars and helicoidal structures.

Silicon single crystals with an (100) orientation and whose resistivity is 5·10^−3^ Ω.cm, or fluorine-doped tin oxide (FTO)-coated glasses, were utilized as substrates depending on the subsequent type of characterization. The substrates were cleaned with detergent solution, rinsed with ultra-pure water, and placed at the ground potential and at ambient temperature prior to deposition.

### 2.2. Characterization Techniques

Field emission gun scanning electron microscopy (FEG-SEM Hitachi SU8020, Hitachi, Tokyo, Japan) was used to observe the microstructure of the nanostructured films, while the nanostructure was investigated by transmission electron microscopy (TEM Philips CM200, Philips, Amsterdam, Netherlands). The cross-sectional lamellae of the untreated nanostructured films were prepared by mechanical polishing and ion milling. Individual columns of the single crystalline thin film were scratched to observe each column separately.

Grazing incidence X-ray diffraction (GIXRD) analysis (Panalytical Empyrean, Malvern Panalytical, Malvern, UK) was used to determine the phase constitution of the samples. The Cu Kα_1_ source (1.5406 Å) was used and the X-ray source voltage was fixed at 45 kV and a current at 40 mA.

The experimental procedure used to design the DSSCs is described in depth elsewhere [35]. Briefly, the dye grafting of TiO_2_-based nano-sculpted electrodes (0.25 cm^2^) was performed by immersion overnight in a solution of acetonitrile and tertbutyl alcohol (volume ratio: 1/1) containing dye sensitizer (0.3 mmol) and (3R),(7R)-dihydroxy-5-cholic acid (Sigma-Aldrich, Saint-Louis, Missouri, MO, USA) (2 mmol) to avoid aggregation of the dye. Then, the sensitized electrode was assembled with a platinized FTO electrode, both separated by 25 μm-thick Surlyn^®^ (Dow Chemical, Midland, Michigan, MI, USA) to prevent the electrolyte from leaking. The internal space was filled with a liquid electrolyte by using a vacuum backfilling system. The photovoltaic performances of the cells were then measured under a simulated air mass (AM 1.5) Global spectrum and 1000 W/m^2^ illumination.

### 2.3. Simulation Protocol

The simulation of nano-sculpted film growth is possible with kinetic Monte Carlo (kMC) algorithms [36,37]. This approach is useful for the modeling of various surface processes such as the nucleation, growth, structural modifications, or dynamic evolution of obliquely deposited structures [38,39]. These features are implemented into the freely distributed NASCAM (nanoscale modeling) code (version 4.6.2 rev. 6; University of Namur: Namur, 2018) [40,41], which is particularly suitable to simulate GLAD processes as it takes into account the motion of the substrate during deposition (translation, rotation, and oscillation).

In this code, the incoming vapor flux is represented by hard spheres and their mobility is simulated according to the ballistic deposition approximation for minimizing the computation time. Taking into account the energy and the angular distribution of the vapor source, those atoms travel toward the substrate along linear trajectories. Then, the deposited particles become part of the growing film.

**Figure 3:**
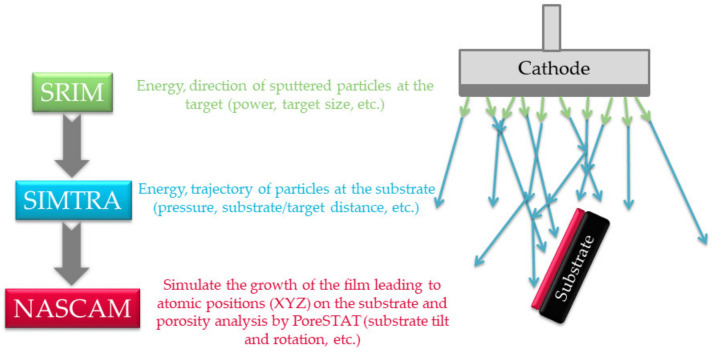
Kinetic Monte Carlo simulation strategy.

As input, the code uses the kinetic energy and angular distribution of the sputtered atoms calculated by SRIM (stopping and range of ions in matter, SRIM-2013; Chester, Maryland, MD, 21619, USA) [42] and SIMTRA (simulation of the metal transport, version 2.2; University of Ghent: Ghent, 2018) codes, respectively. Indeed, the energy and the direction of the particles that are sputtered from the target material are first calculated by SRIM. Then, SIMTRA simulates the transport of these species toward the substrate, taking into account all collisions happening in the gas phase. At the end, the PoreSTAT plugin can be used to evaluate the porosity of the simulated films from the NASCAM output files [43]. This simulation strategy is presented in Figure 3.

The mobility of the atoms that reach the substrate is severely dependent on the energy of incoming deposited atoms. However, the morphology of the films depends on the different deposition parameters [44,45,46,47]. Consequently, to simulate the growth of thin films synthesized at high temperature or with high-kinetic-energy incoming atoms, the approximation of ballistic deposition has to be completed by the diffusion phenomenon [22,27]. Diffusion and evaporation events can take place between two atom depositions at an equal time interval determined by the deposition rate. For each step of the simulation, a list of atoms that can diffuse at the surface or evaporate is created for each possible physical event. The evolution of the system is, thus, determined by the probabilities of the events that may occur during the simulation. This probability can be implemented into NASCAM code via their activation energies (*E*_a_), which can either be found in the literature or calculated by molecular dynamics or potential models.

For each working condition, the energy and the angular distribution of the species can be adapted by the introduction of the experimental parameters such as the working pressure, the power applied to the target, the racetrack size, and the target-to-substrate distance. In order to compare simulated and experimental thin films having the same thickness, the number of deposited atoms (*N*) and the substrate size (*XYZ*) can be tuned in the NASCAM input file. *X* and *Y* correspond to the length and width of the substrate, respectively, while *Z* accounts for the height of the deposited film in atom units.

2D NASCAM simulations were performed for direct comparison with the cross-sectional film morphology, while 3D simulations were performed for the porosity evaluation. The Ti and Mg deposition rates were fixed at 0.5 monolayers per second (0.16 and 0.30 nm/s, respectively), which is of the same order of magnitude in comparison with their experimental values (0.17 and 0.32 nm/s, respectively).

## 3. Results and Discussion

In this section, we overview part of our recent works on the synthesis of nano-sculpted thin films. First, our understanding of the growth mechanisms of these nano-sculpted materials is presented. Then, their utilization in DSSC applications is demonstrated and discussed.

### 3.1. Growth Mechanisms of Metallic Films

**Figure 4:**
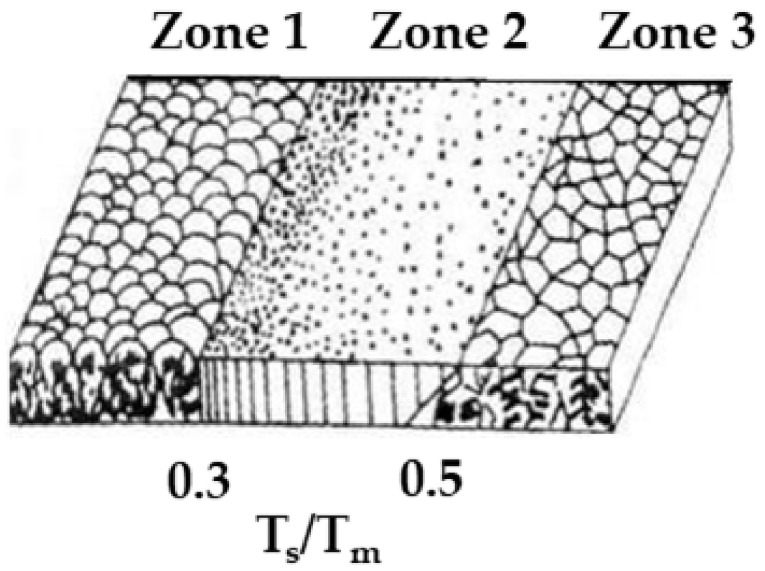
Structure zone diagram of Movchan and Demchishin applicable to the film growth by physical vapor deposition (PVD) (reproduced from [48] with permission from Springer, 2014).

In the first attempt, we establish the deposition parameters, allowing the growth of nano-sculpted metallic thin films by MS-GLAD. Metallic materials were chosen because of their easiest implementation in the NASCAM software. Ti and Mg were chosen as model materials because their growth mechanism is expected to be different according to the structure zone model (SZM) of vacuum-deposited materials published by Movchan and Demchishin [48] (see Figure 4) This model describes the morphologies of the deposited films according to the homologous temperature *T*_s_/*T*_m_, where *T*_s_ is the substrate temperature and *T*_m_ is the melting point of the deposited material. Three zones characterizing different film morphologies, mainly depending on surface diffusion phenomena, are defined as a function of *T*_s_/*T*_m_. Zone 1, corresponding to *T*_s_/*T*_m_ < 0.3, is characterized by a low diffusion and mobility of the adsorbed atoms, leading to a columnar structure with fibrous morphology and weakly bounded grains. For 0.3 < *T*_s_/*T*_m_ < 0.5, Zone 2 is reached and the surface diffusion of the adatoms has a dominant effect on the film growth, leading to a tightly packed columnar grain structure. Finally, Zone 3, obtained for *T*_s_/*T*_m_ > 0.5, corresponds to the condition for which bulk diffusion processes are activated, enabling the diffusion of the grain boundary, as well as the recrystallization during the film growth. For room-temperature growth conditions, *T*_s_/*T*_m_ is 0.15 and 0.32 for Ti and Mg, respectively, which clearly highlights different surface diffusion behaviors during the growth of these materials.

Furthermore, from an application point of view, TiO_2_ is of great interest as the charge transport layer in DSSCs [2], while Mg and its hydride are promising candidates for hydrogen storage [49].

#### 3.1.1. Effect of the Angle of Deposition

**Figure 5:**
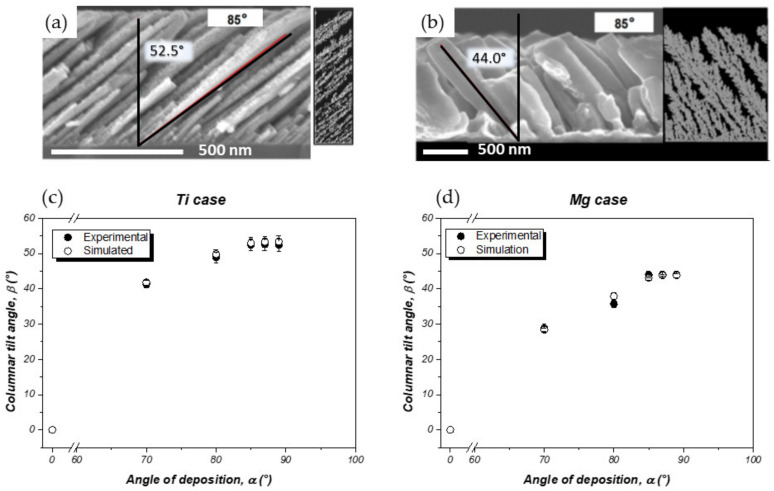
SEM (scanning electron microscopy) cross-sectional view with the corresponding simulation of: (**a**) Ti and (**b**) Mg columnar films deposited with *α* = 85°. Evolution of the columnar tilt angle (*β*) as a function of the angle of deposition (*α*) for (**c**) Ti and (**d**) Mg nano-sculpted thin films. The error bars were estimated by taking the tilt average over 20 columns (adapted from [50] with permission from Elsevier, 2017, and from [32], MDPI, 2019).

The effect of the angle of deposition, α, on the morphological properties of Ti and Mg thin films was first investigated. For this set of experiments, the Ti and Mg films were deposited using high power (150 and 50 W, respectively) and low pressure (0.13 and 0.23 Pa, respectively) in order to generate a ballistic flux by maximizing the deposition rate (~10 and ~19 nm/min, respectively). The thickness of the deposited films was about (500 ± 50) and (620 ± 20) nm for Ti and Mg films, respectively. Ti and Mg thin films synthesized for various α angles from normal incidence (*α* = 0°) to grazing angle (*α* = 89°) with the corresponding kMC simulations are shown in Supplementary Materials, Figures S1 and S2. A number of 1.2 × 10^5^ and 5 × 10^5^ atoms was chosen for similar experimental and simulated film thicknesses according to the size of the Ti (*X* = 250 and *Y* = 2 Ti atom units) and Mg (*X* = 1000 and *Y* = 2 Mg atom units) simulation boxes, respectively. The evolution of the morphological properties assessed by the analysis of the cross-sectional SEM (scanning electron microscopy) images, as well as by the NASCAM simulation tool for both Ti and Mg nano-sculpted materials as a function of *α*, is presented in Figure 5.

As expected, the increase in α leads to generation of a nano-sculpted thin film composed by well-separated tilted columns in both cases (Ti and Mg materials). As explained in the introduction part, the columnar structure is strongly influenced by shadowing effects, particularly at extreme oblique incidence angles (>60°). If many features are common between both systems, we can notice that the width of the columns is higher for Mg than for Ti (see Figure 5a,b). This is explained by the higher homologous temperature for the Mg deposition; ~0.35 vs. ~0.19 for Ti, which means that the growth of Ti thin films belongs to the Zone 1 regime, while the growth of Mg thin films belongs to the Zone 2 regime of the SZM, allowing surface diffusion of the Mg adatoms, which ultimately leads to less dense and thicker columns compared to the Ti case.

Figure 5c,d shows that, for both metals, the columnar tilt angle (*β*) and the inter-columnar space drastically increase in these conditions. However, the *β* value stabilizes for *α* ≥ 85°, whatever the considered metal. This observation can be explained by the specific geometry of our experimental setup. Indeed, the diameter of the target (5 cm) has to be taken into account to distinguish α from the incident angle of the particles as the substrate-to-target distance (8 cm) is not very high in our geometry. Hence, the majority of the deposited particles comes from the racetrack region of the target. The size of the particles source thus induces a deviation in the *α* or *ф* directions corresponding to the angular distribution of the particles reaching the substrate, and increases with the target diameter. The width of the sputter flux distribution at 0.13 Pa, considering the geometry we used, was determined by using SIMTRA, and the angle of deviation was simulated to be around 10° (see Supplementary Materials, Figure S3). A stabilization of the *β* value for *α* ≥ 85° was observed because the geometrical inclination of the substrate leads to an asymmetric deposition, and the particles sputtered at the left side of the target have a higher probability to reach the left side of the substrate compared to those sputtered on the right side.

All these behaviors are in almost perfect agreement with the simulated data, as shown in Figure 5c,d, which demonstrate the relevance of the modeling approach that is utilized. From these data, it is finally possible to highlight the importance of surface diffusion in the generation of such nano-sculpted metallic films.

On the other hand, a systematic lower *β* value in comparison with *α* is observed in all situations for both metals. This might be understood by the parallel momentum (kinetic energy) conservation principle [51]: under oblique incidence, the amount of kinetic energy conserved in the direction parallel to the film surface is determined by the angle of incidence. Thus, *β* varies with α and the kinetic energy of the impinging vapor atoms.

Based on these initial works, *α* = 85° was chosen for all subsequent thin-film syntheses as it allows well-defined columnar structures to be produced at reasonable deposition rates.

#### 3.1.2. Effect of the Deposition Pressure

The deposition pressure (*P*_dep_) is another important parameter in GLAD experiments. In this part, *P*_dep_ was varied from 0.13 to 1.3 Pa, while the sputtering power was fixed at 50 W for Mg and 150 W for Ti, and α was fixed at 85° in both cases. As this parameter does not influence the surface diffusion of the adatom, a similar dependence is observed for Ti and Mg. Therefore, we will only discuss the results obtained for the Ti films. A detailed description of the Mg case can be found elsewhere [32], and the cross-sectional SEM images associated with the simulated data are presented in the Supplementary Materials, Figure S4.

**Figure 6:**
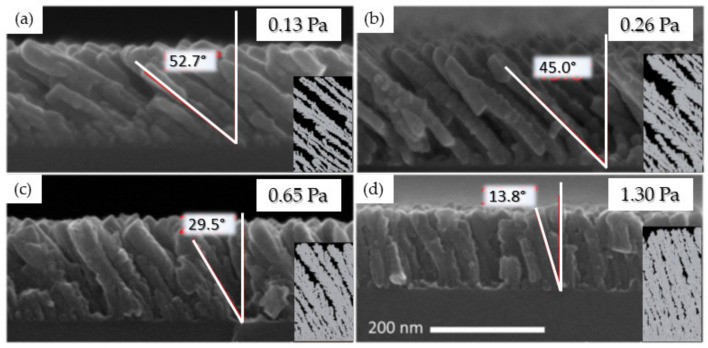
Ti thin films synthesized at 150 W and 85° for various deposition pressures: (**a**) 0.13; (**b**) 0.26; (**c**) 0.65; (**d**) 1.3 Pa with the corresponding simulations. The red angle accounts for the average columnar tilt angle (*β*) estimated by over 20 columns (adapted from [50] with permission from Elsevier, 2017).

From the data shown in Figure 6, we learn that *β* rapidly decreases as *P*_dep_ increases, from (52.7 ± 2.0)° for 0.13 Pa to (13.8 ± 1.9)° for 1.3 Pa. As the collision probability increases with *P*_dep_, this is attributed to a decrease in the collimation of the incident particle flux. Indeed, this probability is expressed by the mean free path of the sputtered atoms (*λ*) and is inversely proportional to *P*_dep_ as follows:

(1) $$\lambda =\;\frac{k_{B}T}{\sqrt{2}\pi d^{2}P_{\mathrm{dep}}}$$

where $k_{B}$ is the Boltzmann constant, T is the temperature in K, *P*_dep_ is the pressure in Pa, and d is the diameter of the gas particles in m [52]. Considering the atomic diameter of Ti particles (1.4 Å), *λ* ranges from 45 to 4.5 cm between 0.13 and 1.3 Pa, respectively. Owing to the target-to-substrate distance used in this work (8 cm), an increase in *P*_dep_ to 1.3 Pa induces a large amount of collisions between particles, resulting in a divergent particle flux reaching the substrate, and the film becomes denser.

**Figure 7:**
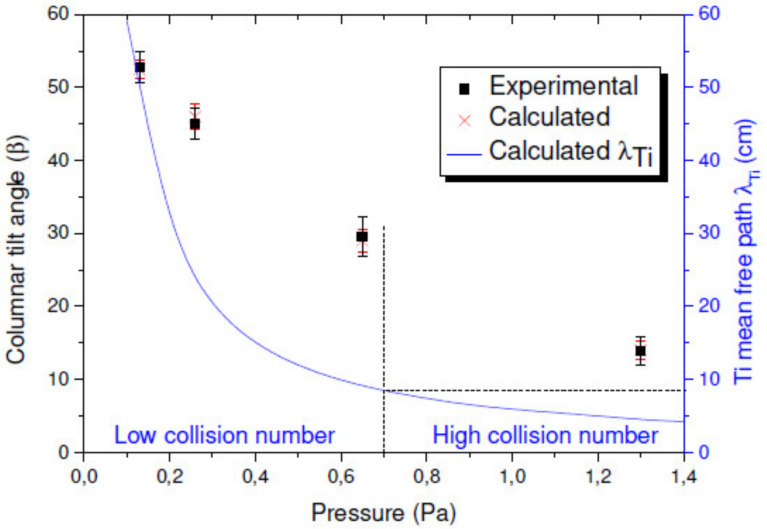
Columnar tilt angle as a function of the deposition pressure for experimental and simulated thin films. The blue line corresponds to the evolution of the mean free path of the sputtered Ti atoms calculated from Equation (1) (reproduced from [50] with permission from Elsevier, 2017).

The comparison between experimental and calculated values of *β* as a function of *P*_dep_, as well as the calculated *λ* value for Ti atoms, is shown in Figure 7. The critical *P*_dep_ value from which *λ* becomes smaller than the target-to-substrate distance is estimated as ~0.7. Very few collisions occur through the vapor phase below this value, while numerous collisions between particles occur at higher pressure.

SIMTRA calculations support this hypothesis as the simulated number of collisions between particles increases from 0.7 to 25 when increasing *P*_dep_ from 0.13 to 1.3 Pa. Furthermore, the angular distribution of the incoming Ti atoms has a low standard deviation (<30°) at 0.13 Pa, while it presents a large deviation (>100°) at 1.3 Pa [50].

Based on these analyses, the range of pressures where a ballistic deposition process occurs is, in our situations, from ~0.13 and ~0.26 Pa.

#### 3.1.3. Effect of the Substrate Temperature

The substrate temperature (*T*_s_) is one of the most important parameters influencing the growth mechanism of MS-GLAD thin films as the mobility of the adatoms, which is demonstrated to be a key element, can be activated through many diffusion phenomena by increasing *T*_s_.

**Figure 8:**
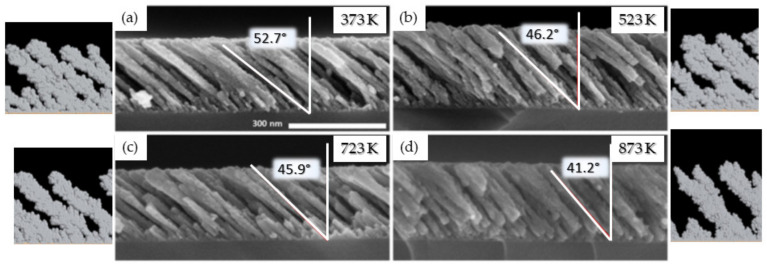
Ti thin films synthesized at 150 W, 0.13 Pa, and 85° for various substrate temperatures: (**a**) 373; (**b**) 523; (**c**) 723; (**d**) 873 K with the corresponding simulations. The red angle accounts for the average columnar tilt angle (*β*) estimated by over 20 columns (adapted from [50] with permission from Elsevier, 2017).

Tilted columnar Ti thin films were synthesized for 373 K < *T*_s_ < 873 K, where 373 K corresponds to the substrate surface temperature without intentional heating. This value is higher than the ambient temperature because of energy transfer phenomena occurring at the plasma-growing film interface associated with the IR radiation emanating from the Ti target [27]. The sputtering power was fixed at 150 W while *P*_dep_ = 0.13 Pa and *α* = 85°. Figure 8 shows that by increasing *T*_s_, *β* decreases from (52.7 ± 1.8)° to (41.2 ± 1.5)°. This can be understood according to the SZM diagram discussed in Section 3.1 (see Figure 4). Indeed, based on this diagram, we learn that the films synthesized for *T*_s_ up to 523 K belongs to Zone 1 (*T*_s_/*T*_m_ ≤ 0.27), meaning that the surface diffusion is limited, allowing for the formation of porous and well-defined columnar morphologies. The geometric configuration governs the formation of microstructures in these conditions, leading to a strongly anisotropic deposition with a low influence of *T*_s_ on *β*. However, for 723 K < *T*_s_ < 873 K (i.e., 0.37 < *T*_s_/*T*_m_ < 0.45), the films belong to Zone 2 of the SZM diagram. In that case, the anisotropic character of the deposited films is induced by the activation of surface diffusion, leading to a decrease in *β*.

In order to simulate the growth of nano-sculpted films at high temperature, the approximation of ballistic deposition, therefore, has to be completed by diffusion phenomena. The physical mechanisms that can be thermally activated, such as diffusion on a substrate and hopping from a substrate on an island, behave according to the following exponential law:

(2) $$w_{i}=w_{0}\;\exp\left(\frac{-\Delta E_{a}}{k_{B}T}\right)$$

where $w_{0}$ is the attempt rate that can be estimated as $w_{0}=2k_{B}T$, and $\Delta E_{a}$ is the activation energy for the physical mechanism.

Based on many sets of simulations, it appears that the critical events of diffusion that affect the morphology of the films synthesized by GLAD are the hop up and hop down from one atomic layer to another (*E*_a,up_ and *E*_a,down_, respectively). The values leading to a better agreement with the experimental data are 2.0 and 2.5 eV for *E*_a,up_ and *E*_a,down_, respectively, while *E*_a,diffusion_ is fixed at 1.35 eV. Finally, the low Ti vapor pressure (10^−4^ Pa at 1783 K [53]) strongly limits the probability that an evaporation event occurs in the temperature and pressure conditions used in this work, and is lower than a diffusion event. Consequently, *E*_a,evap_ was fixed at 5.0 eV.

Simulations were, thus, performed for the same conditions than for the experiments to evaluate the impact of the diffusion phenomena on the film morphology (see Figure 8). The agreement between the experimental and simulated data validate that the diffusion is the reason for the evolution of *β* with *T*_s_. Based on that, it is possible to adapt the empirical model proposed by Movchan and Demchishin for thin films deposited at oblique angles with different *T*_s_ [50].

**Figure 9:**
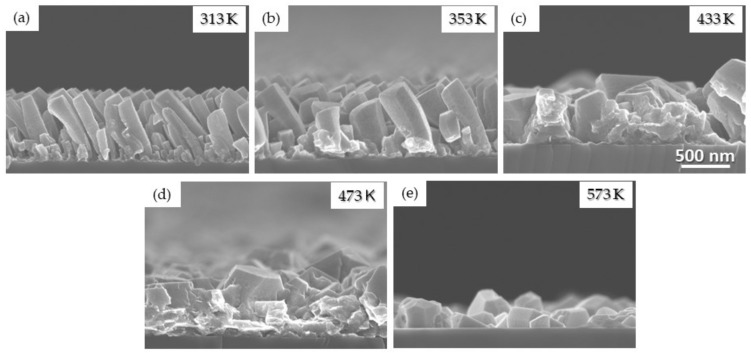
Mg thin films synthesized at 50 W, 0.26 Pa, and 85° for various substrate temperatures: (**a**) 313; (**b**) 353; (**c**) 433; (**d**) 473; (**e**) 573 K.

Similar experiments were performed for the Mg case by varying *T*_s_ between 313 and 573 K with the other experimental conditions as follows: *P* = 50 W, *P*_dep_ = 0.26 Pa, and *α* = 85°. As previously discussed, Mg is a high-mobility material as its melting point is low enough to allow a Zone 2 growth mechanism at room temperature. The cross-sectional SEM images are presented in Figure 9.

The films grown for *T*_s_ = 313 and 353 K, corresponding to a homologous temperature of ~0.34 and ~0.38, respectively, reveal a columnar structure with a decrease in *β* and a larger columnar width when increasing *T*_s_, which is a similar behavior to Ti. For higher temperature, the columnar structure is lost and faceted grains appear. This is due to the activation of the bulk diffusion processes leading to bigger and recrystallized grains, as predicted by the SZM diagram in the Zone 3 regime that is reached in these conditions (*T*_s_/*T*_m_ ~ 0.51 and 0.62, respectively). Figure 9c represents the intermediate situation (*T*_s_/*T*_m_ ~ 0.47) where a weak columnar structure with large grains and reduced inter-columnar spaces can still be observed.

#### 3.1.4. Effect of the Substrate Rotation

**Figure 10:**
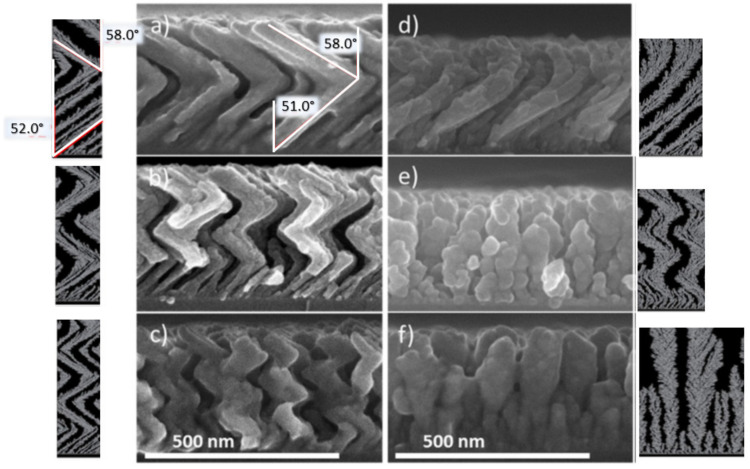
Ti thin films (150 W, 0.13 Pa, 85°) deposited at various rotations of the substrate to generate: (**a**–**c**) Zigzag structures; (**d**) helicoidal structures at 0.1°/s; vertical pillars at (**e**) 1.0°/s and (**f**) 10.0°/s with the corresponding simulations. The red angle accounts for the average columnar tilt angle (*β*) (adapted from [50] with permission from Elsevier, 2017).

Using the optimal conditions established above (low *T*_s_ and *P*_dep_, namely 373 K and 0.13 Pa, respectively), the effect of the rotation angle (*ф*) on the microstructure of the Ti film was evaluated. As a reminder, a variation in *ф* (+180° or −180°) leads to zigzag structures with various numbers of branches depending of the number of cycles, while vertical or helicoidal structures are generated for a continuous rotation of the substrate (0.1, 1.0, or 10.0°/s), as shown in Figure 10.

At the bottom of the images, we can observe the initial stages of the growth including a large number of small objects. Then, the number of nano-objects rapidly decreases due to the competitive growth occurring, while the film becomes thicker. This effect is more pronounced by the modification of substrate orientation during the generation of zigzag structures, which also induces a variation in *β* (see Figure 10a). The rotation of the substrate with *ф* modifies the local deposition geometry. The first nuclei are deposited with a uniform *α* onto a flat substrate, allowing a corresponding *β*. After that, the substrate is rotated at 180° and the incident vapor thus meets a surface with a different orientation because deposition then takes place on top of the first object. The effective local deposition angle *α*’ that occurs during the deposition with α onto a tilted column with *β* is then *α*’ = *α* + *β* − 90° [54].

The corresponding simulated data are obtained without taking into account diffusion phenomena. The evolution of the growth, as well as the final microstructure (*β* variation between two zigzags), are reproduced well. The inter-column competition, explaining the decrease in the number of nano-objects during the film growth, starts during the nucleation stage. Hence, the evolution of the columnar growth is driven by this competition mechanism and generates a film morphology scale-invariant, in view of the different stages of the growth [55].

#### 3.1.5. Evolution of the Porosity

**Figure 11:**
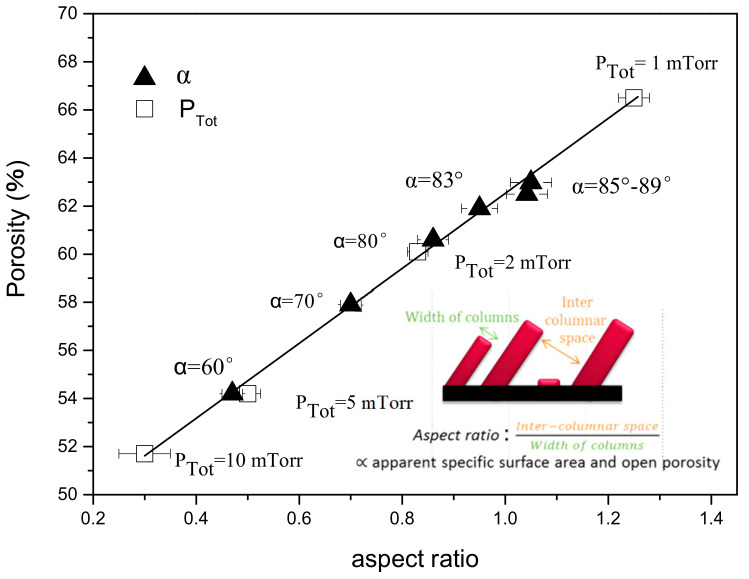
Evolution of the porosity as a function of the aspect ratio for Mg nano-sculpted films deposited at various deposition pressures and *α*. The inset illustrates the definition of the aspect ratio, *Г* (adapted from [32], MDPI, 2019).

In order to obtain an evaluation of the porosity of our films, a NASCAM simulation has been used. This is one of the most important outputs of these simulations as it is very difficult to experimentally measure such a kind of porosity in thin-film materials. To do this, the output data of the NASCAM simulations were treated using the PoreSTAT plugin, which basically defines the size of the pores in order to evaluate the porosity (*Ф*) of the generated structures. In the case of Mg thin films, we observed a linear evolution of *Ф* with the so-defined aspect ratio (*Г*), which corresponds to the ratio of the inter-columnar spaces on the width of the columns (see Figure 11), assessed from electron microscopy images. This parameter gives an indication of the specific surface area of the material. For example, more space between the columns or a reduction in the column width will lead to an increase in the material porosity, and thus, of *Г*. Therefore, the tuning of the key parameters during the growth such as *P*_dep_ and *α* allows the film porosity to be finely monitored, which is very interesting in view of the applications that are foreseen.

**Figure 12:**
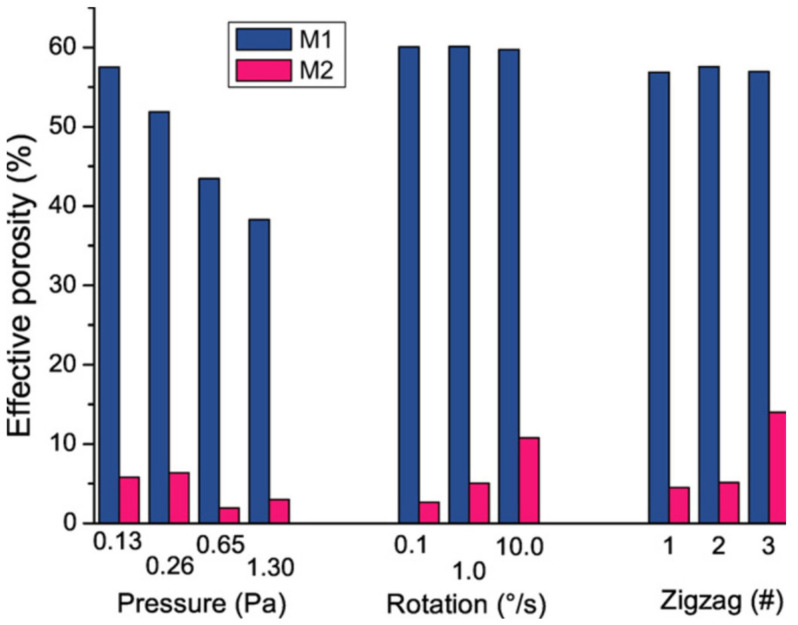
Kinetic Monte Carlo (kMC) tri-dimensional (3D) analyses of the effective porosity for different conditions with pore sizes above 0.64 nm (M1) and above 3.2 nm (M2) (reproduced from [50] with permission from Elsevier, 2017).

For Ti thin films, similar simulations have been performed with even more details. Indeed, in this case, the porosity has been computed as an “effective porosity,” *Ф*_e_, because the porosity value depends on the size of the object that could penetrate the system. To do this, two molecules (M1 and M2) having different diameters (0.64 and 3.20 nm, respectively) have been considered to penetrate the Ti nano-sculpted films. The calculated ratio between the total porosity volume and the accessible porosity for M1 and M2 molecules penetration gives the *Ф*_e_ for each molecule. This gives a flavor of the organization of the porosity at the nanoscale. Many models have been performed for different types of Ti nano-sculpted films. Figure 12 summarizes the evolution of *Ф*_e_ with various experimental parameters for M1 and M2 molecules.

**Figure 13:**
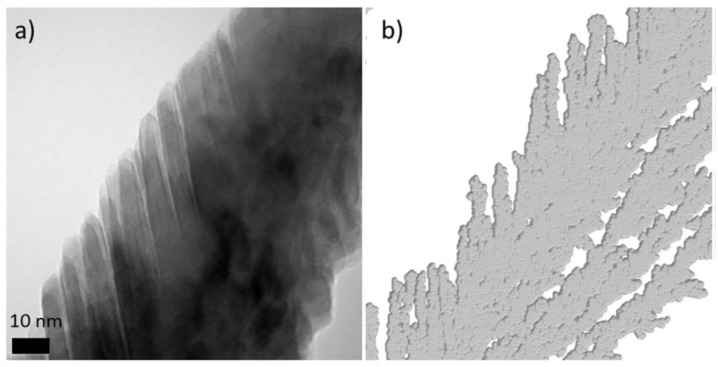
(**a**) Transmission electron microscopy (TEM) image of a Ti micro-column constituted by nano-columns from a Ti thin film synthesized at 0.13 Pa, 150 W, and 85°; (**b**) the corresponding kMC simulation where each atom is represented by its covalent radius (reproduced from [50] with permission from Elsevier, 2017).

A significantly higher *Ф*_e_ value is obtained for the smallest molecule in all studied conditions: Around 60% for M1 vs. less over 5% for M2. This can only be explicated by the nanoscale structure of the film, suggesting a hierarchical growth, as observed in both TEM image and 2D simulation (see Figure 13): large micro-columns are formed by the agglomeration of the nano-columns upon increase in the film thickness. This is especially the case in low-mobility deposition conditions. Since then, the open-porosity from the nano-columns has not been available for the grafting of large molecules such as M2.

The results presented in Figure 12 also revealed that: (i) high *P*_dep_ induces low *Ф*_e_ values, which is associated with the already discussed densification of the film, while (ii) the largest *Ф*_e_ values are obtained with a high substrate rotation speed or high number of zigzags. The latter behavior can be understood by the fact that the inter-columnar spaces become larger when a reduced number of growing columns are promoted because of the modification of the substrate orientation during deposition. Furthermore, the *Ф*_e_ associated with the M1 molecule is quite constant because of its small size. Indeed, M1 might impregnate the film even without variation in *ф*. Consequently, increasing the inter-columnar space by modifying *ф* does not influence the *Ф*_e_ value of sufficiently small molecules.

### 3.2. Nano-Sculpted Oxide Films

From our work on metallic films, we learn that the melting temperature and the associated reduced temperature is a key element, allowing the structure of nano-sculpted films synthesized by MS-GLAD to be understood. Therefore, we expected a different impact for TiO_2_ and MgO thin films compared to their respective metallic form as the melting point of TiO_2_ is close to that of Ti (~2116 vs. ~1941 K, respectively), while for MgO, the difference is important (~3125 vs. ~923 K for Mg). For both Ti and Mg cases, therefore, we synthesized the corresponding oxides by adding O_2_ to the RMS-GLAD process. Detailed descriptions of these studies can be found in [9] and [31], respectively.

**Figure 14:**
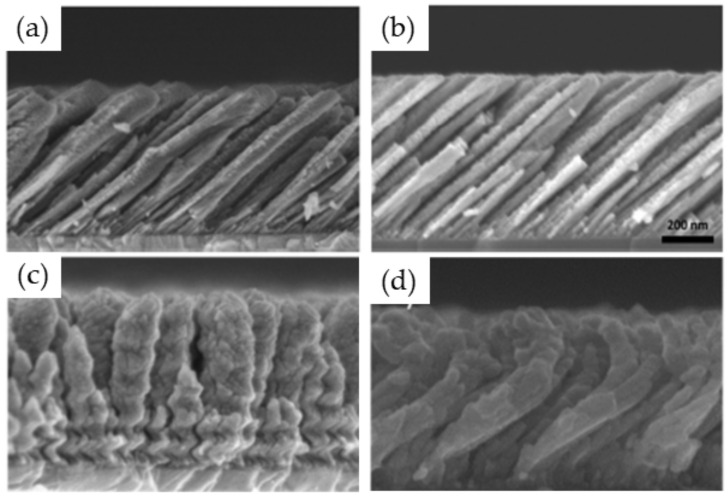
Cross-sectional SEM images of nano-columnar (**a**) TiO_2_ and (**b**) Ti thin film deposited at 150 W, 0.13 Pa, and *α* = 85°. Cross-sectional SEM images of helicoidal (**c**) TiO_2_ and (**d**) Ti thin film deposited at 150 W, 0.13 Pa, *α* = 85°, and *ф*_s_ = 0.1°/s (adapted from [50] and [9] with permission from Elsevier, 2017 and 2015, respectively).

As expected from the difference in melting points between Ti and TiO_2_, we do not observe significant differences from cross-sectional SEM images between the morphologies of both nano-sculpted films prepared for similar conditions (Figure 14a,b). Indeed, in both cases, *T*_s_/*T*_m_ belongs to the Zone 1 regime (~0.19 vs. ~0.18, respectively). This suggests that our understanding of the growth of nano-sculpted thin films by RMS-GLAD based on the value of *T*_s_/*T*_m_ is adapted independently of the chemistry of the studied compound. This observation is further supported by experimental data, revealing that self-diffusion from the bulk to the surface is initiated for temperatures higher than 400 K in TiO_2_ [56]. This temperature is higher than the *T*_s_ value reached without intentional heating in our work (~373 K). We can, therefore, conclude that the growth mechanisms for Ti and TiO_2_ are very close and that, as a consequence, the generated nano-sculpted films are similar. This is important as the simulation approach employed in our work is not yet adapted to the growth of oxide materials and, therefore, cannot be used to predict the morphology of such materials.

Nevertheless, this conclusion has to be moderated in certain situations such as that of pillar films shown in Figure 14c,d. In this case, because of the significantly lower deposition rate of the materials (2 vs. 10 nm/min for TiO_2_ and Ti, respectively), the number of turns of the helical column increases consistently with the rotation speed, resulting in an elongated morphology [57]. Consequently, TiO_2_ films have an elongated morphology by reducing the helical pitch (i.e., the height of one turn of a helix) due to the lower deposition rate, for similar rotation speed. However, the rotation speed limitation of the substrate holder (0.1°/s) does not allow the synthesis of films, presenting a morphology with large helicoidal pitches as that observed for Ti.

**Figure 15:**
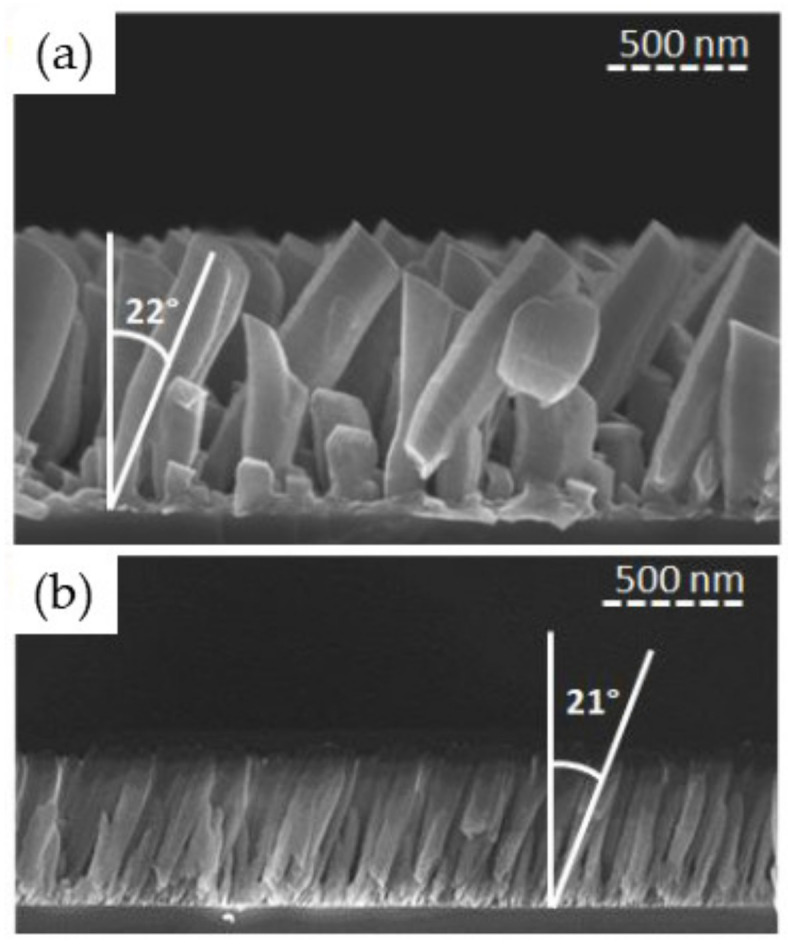
Cross-sectional SEM images of nano-columnar (**a**) Mg and (**b**) MgO thin film deposited at 50 W, 0.26 Pa, and *α* = 85°. The white angle accounts for the average columnar tilt angle (*β*) estimated by over 20 columns.

As expected from the melting temperature difference between Mg and its corresponding oxide MgO, the story is different in this case. It indeed appears that the morphology of the sample is strongly affected by the addition of O_2_ in the gas mixture (see Figure 15). The features of the columnar structures, i.e., the column width and the inter-columnar space, are reduced from (171 ± 18) to (56 ± 11) nm and from (120 ± 25) to (37 ± 16) nm, respectively, when increasing the flux of O_2_ added to the discharge. Furthermore, the shape of the single columns is affected as well because pure Mg columns are strongly faceted, while they are no longer faceted when a small amount of O_2_ is added to the discharge. However, *β* remains stable beyond the transition.

The modification of the chemistry of the deposited material actually impacts the diffusion kinetic of the growing material on its substrate. Indeed, in this case, *T*_m_/*T*_s_ is significantly reduced for the MgO situation when compared to the Mg case (~0.10 vs. ~0.35, respectively). The deposition of MgO nano-sculpted films, thus, belongs to the Zone 1 regime of the SZM while the deposition of Mg belongs to the Zone 2 regime, as described above. This leads, for MgO, to a denser population of thinner grains acting as starting sites for the columns growth, thus explaining the evolution of the film microstructure in reactive conditions.

### 3.3. Growth Model for GLAD Deposited Thin Films

**Figure 16:**
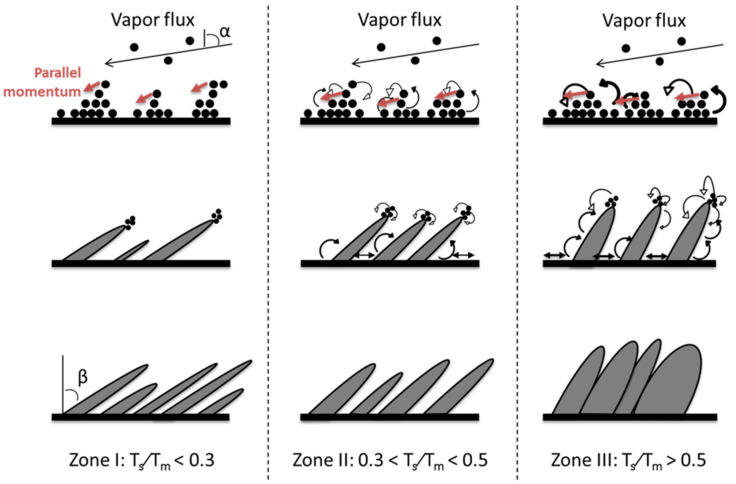
Growth models expected for oblique angle deposition at different substrate temperatures (reproduced from [50] with permission from Elsevier, 2017).

Based on the aforementioned studies, it is possible to adapt the empirical model for thin films deposited at normal incidence (*α* = 0°), proposed by Movchan and Demchishin, to deposition in an oblique angle configuration with different *T*_s_ (see Figure 16).

As already mentioned, the parallel momentum affects *β*, especially when the diffusion process is favored as it is the case under oblique incidence. In addition to that, the diffusion is also enhanced during deposition at high temperature. Thus, the homologous temperature limits of the adapted growth model for thin films under oblique incidence are the same as the normal incidence case.

In Zone 1 (*T*_s_/*T*_m_ < 0.3), the diffusion process hardly takes place and the incident particles generally stay in the original deposition site, leading to a high number a thin columns producing small and numerous inter-columnar spaces.

For 0.3 < *T*_s_/*T*_m_ < 0.5, Zone 2 is reached, allowing hops up and hops down events after accommodation, until adatoms reach an “energetically favorable” site. The number of columns will, thus, decrease but become thicker because of the agglomeration process.

A further increase in *T*_s_ finally allows Zone 3 (*T*_s_/*T*_m_ > 0.5) to be reached. The diffusion processes are increasingly enhanced and are now almost perpendicular to the substrate surface. Thus, the adatoms are able to reach the shadowed area, leading to the coalescence of the columns. The films porosity is thus highly reduced. More information about the time before accommodation can be found elsewhere [50].

### 3.4. Nano-Sculpted TiO_2_ Films for Dye-Sensitized Solar Cell Applications

If the fundamental understanding of the observed phenomenon is the key element motivating our efforts, the applications of the developed materials in a meaningful field such as that of energy-related technologies are equally important. Therefore, the developed nano-sculpted thin films have been employed for energy-related applications such as for the design of novel hydrogen storage materials [32] or of novel photoanodes in DSSCs. The most important results obtained for the latter case will be summarized in the following pages.

#### 3.4.1. Context

Considering current global concerns about the increase in energy consumption, the depletion of deeply used fossil fuels associated with the consequences of global warming makes the efficient use of renewable energies a major economic and environmental interest. Among all the studied renewable energy-based technologies, solar energy is definitely the most promising of them because of its abundance. In this context, dye-sensitized solar cells (DSSCs) are recognized as a potential low-cost photovoltaic solution owing to its many attractive advantages: Cheap production cost, tunable transparency, aesthetic capability, lightweight devices, but most importantly, good performances under low-illumination and high-temperature conditions [2,58,59].

**Figure 17:**
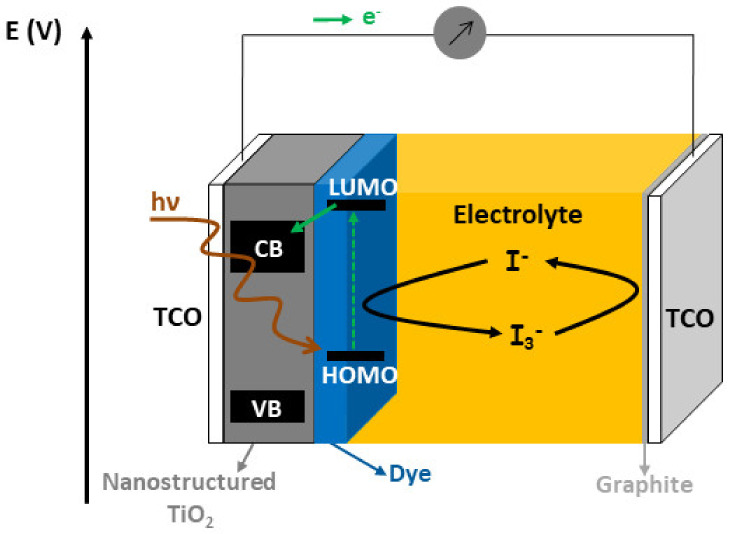
Schematic electron transport pathway in dye-sensitized solar cells (DSSCs).

The conventional architecture of a DSSC involves three key components: A porous array of n-type semiconductors (classically sintered TiO_2_ nanoparticles), on which an electron-donor dye is adsorbed, both forming the photo-anode. The role of the dye is to absorb solar photons that causes its excitation, followed by electron injection into the conduction band of the semiconductor. Finally, the oxidized dye is regenerated by a redox liquid electrolyte, and the semiconductor transports the electron from the interface with the dye to the electrode [2,58,60]. The liquid electrolyte solution is then reduced at the counter electrode by electrons coming from the photo-anode via the external circuit. A scheme representing the different layers of DSSCs is shown in Figure 17.

In the first development of this technology in 1991, the efficiency was about 7–8% [2]. Most of the shortfall was due to the weak absorption of low-energy photons by common dyes and to the high rate of electron–hole recombination at the semiconductor/dye and/or semiconductor/electrolyte interfaces. Hence, molecular engineering of the dyes and the development of new electrolyte mediators associated with the optimization of the device fabrication have enabled us to reach solar-to-electricity conversion efficiencies of 14% at the highest [61,62,63,64]. In addition, it is also accepted that the semiconductor composition and morphology strongly impact the performances of DSSCs, as recently reviewed by Maçaira et al. [65]. TiO_2_ is considered nowadays as the best material because it is abundant, cheap, nontoxic, very stable under visible-light irradiation, and characterized by a wide bandgap, providing a high transmittance in the near UV-visible light region [66,67]. However, the conventionally used TiO_2_-sintered nanoparticles electrodes are known to limit the charge transport by the structural disorder at the nanoparticles boundaries, enhancing electron scattering. This highly reduces the charge collection at the photo-anode, therefore limiting the current provided by the solar cell [9].

Regarding the morphology, as well as the composition of the semi-conductor, being of major interest to allow a high dye uptake, as well as optimized charge transport efficiency, the recent advances performed by the use of nano-sculpted TiO_2_-based photo-anodes is discussed in the following sections.

#### 3.4.2. Nano-Sculpted TiO_2_ Thin Films as Photoanode Material

In order to assess the potential of all morphologies that can be generated by RMS-GLAD, their respective aspect ratio, *Г* (see Figure 11 for the definition), was evaluated from the analysis of SEM pictures. As expected, *Г* depends on the film morphology: A dense film presents a 0 value while the largest value (0.3) is calculated for the slanted columnar morphology (SCM). According to the *Г* parameter, the SCM morphology appears, therefore, to be the best candidate for optimizing the dye uptake capability [68]. This has been verified by assessing the desorption of dye to evaluate the effective specific surface area of the different films. Ru complex dye N719 was used as it allows a reversible grafting on the films. After dye absorption by dip coating of the thin films with fixed surface and thickness, the films were rinsed and immersed in a KOH solution in order to desorb the dye. The absorbance of the resulting solution was thus measured by UV-vis spectrophotometry to ultimately calculate the dye concentration using a calibration procedure. The SCM films allow the highest absorption in agreement with the expectations from the Г values. The highest specific surface area of 86 m^2^/g was obtained for films deposited with *α* = 85°, while the optimized film thickness was evaluated at 3.5 µm with a surface area enhancement around 200 m^2^_N719_/m^2^_substrate_ [68].

The crystalline structure of the deposited films was investigated by X-ray diffraction, which reveals an anatase phase for all generated morphologies. To obtain space-resolved information, electron diffraction during TEM experiments was performed, showing that the films are not homogeneously crystallized: An amorphous phase is deduced at the substrate interface while the anatase phase was detected near the surface region. This late crystallization at the end of the film growth can be explained by a gradual heating of the substrate by ions bombardment, surface reactions, and intense IR radiation emanating from the target [27].

**Figure 18:**
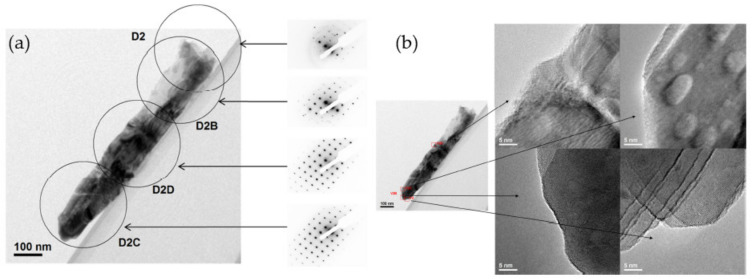
High-resolution TEM picture of an isolated column from a slanted columnar TiO_2_ thin film annealed 2 h at 773 K; (**a**) the corresponding electron diffraction patterns; (**b**) high-magnification pictures at various locations.

In view of the utilization of these films in DSSCs, a homogeneous crystallization of the material is necessary. The usual strategies allowing us to improve the crystalline quality of RMS-deposited films have, therefore, been evaluated, namely biasing the substrate, the substrate heating during the deposition, and the post-annealing of the synthesized films. The data, fully described in [9], revealed that a substrate polarization allows the control of the crystalline phase from pure anatase to pure rutile, but leads to the densification of the thin films while both heating procedures allow a better crystallization into the anatase phase without significantly affecting the film morphology (see Figure 18).

**Figure 19:**
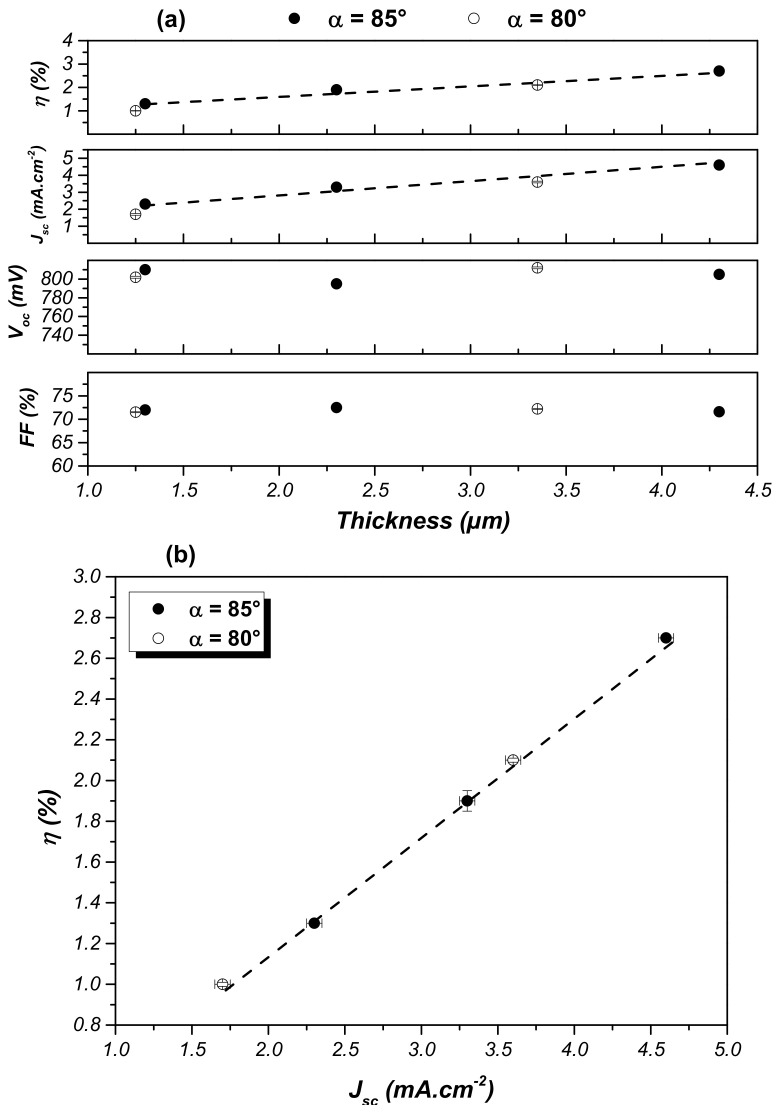
(**a**) Photovoltaic performances of liquid DSSCs integrating a slanted columns-based TiO_2_ film as the photo-anode and according to the thickness of the latter; (**b**) plot of the cell efficiency according to the corresponding *J*_sc_.

These films have then been utilized as the photo-anode in DSSCs. SCMs synthesized for different α and thicknesses were investigated. A reference photo-anode based on conventional TiO_2_ nanoparticles powder was systematically built to validate the utilization of our thin films. The photovoltaic parameters, i.e., the fill factor (FF), open-circuit voltage (*V*_oc_), short-circuit current (*J*_sc_), and efficiency (*η*), are presented in Figure 19.

First, FF and *V*_oc_ parameters are constant around 72% and ~800 mV, respectively. These observations are not surprising, as the combination of MS and GLAD allows well-adherent and -ordered sculpted TiO_2_ thin films to be generated onto the FTO layer, ensuring a good contact. All photo-anodes were post-annealed under an identical procedure, leading to the same crystalline structure.

Furthermore, Figure 19b reveals that the cell efficiency linearly evolves with the current density produced by the cell whatever the angle of deposition. This means that the quantity of adsorbed dye through the corresponding *J*_sc_ is mainly responsible for the overall efficiency of our DSSCs. However, the reference cell based on TiO_2_ nanoparticles is characterized by the relatively same FF and *V*_oc_ (69% and 752 mV, respectively), while the *J*_sc_ and *η* are around 20 mA·cm^−2^ and 10.7%, respectively, meaning that *J*_sc_ is the parameter responsible for the four-times-higher efficiency. Consequently, the slanted columnar morphology allows good charge transport while the density of adsorbed dye remains the critical parameter [35].

#### 3.4.3. A Nano-Sculpted TiO_2_/TiO_2_ Nanoparticles Hybrid Approach

**Figure 20:**
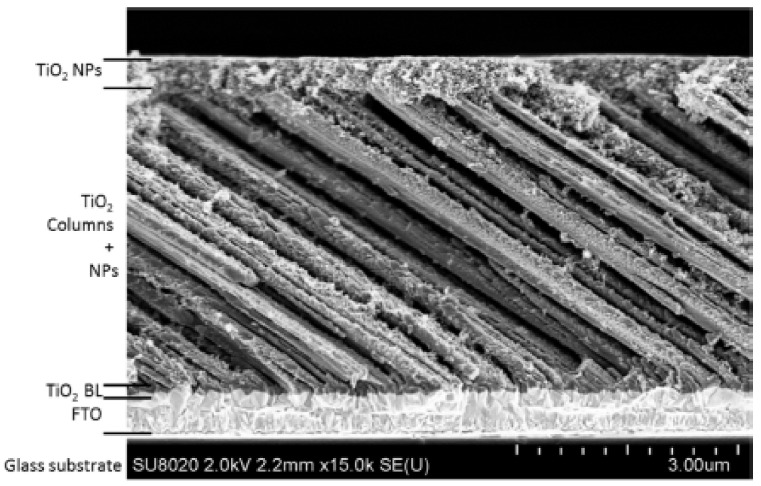
Cross-sectional SEM picture of a slanted columnar thin film (4.3 μm) after spin coating by a TiO_2_ nanoparticles solution.

The easiest way to increase the dye absorption density would be to use thicker photo-anodes. Nevertheless, this option is limited by the RMS process, which allows for the synthesis of less than ~1 µm films typically. On the other hand, the low deposition rate of the nano-sculpted films (~2 nm/min typically) would also be a limiting factor. Therefore, as an alternative, it has been decided to impregnate the nano-sculpted TiO_2_ films with a solution of anatase TiO_2_ nanoparticles (Solaronix^®^, Aubonne, Switzerland) ~20 nm by spin coating. In this way, a hierarchical structure is formed and the voids between the columns are exploited to ultimately increase the dye molecule absorption by the nanoparticles, allowing for a better dye uptake. A cross-sectional SEM image of this hybrid system is shown in Figure 20.

**Figure 21:**
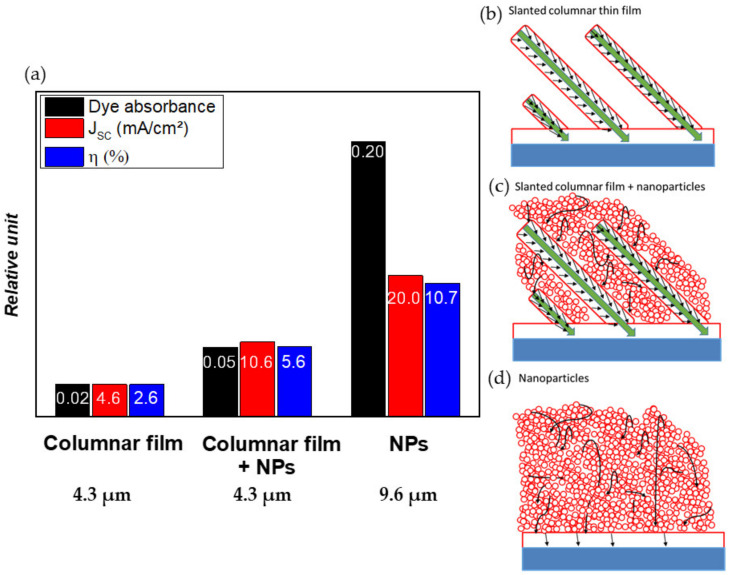
(**a**) Photovoltaic performances of DSSCs based on various photo-anode architectures. Schematic representation of the electron transfer occurring in photo-anode based on: (**b**) Slanted columnar thin film; (**c**) the combination of slanted columns and nanoparticles; (**d**) nanoparticulate thin films.

The photovoltaic performances of the DSSCs based on this hybrid photo-anode have been evaluated, and the results are presented in Figure 21a. First, it appears that the incorporation of NPs significantly increases the absorbance of the film to a value of 0.0518 in comparison with the value of 0.0243 for the SCM thin films (4.3 μm) alone. The incorporation of NPs significantly improves the *J*_sc_ value from 4.6 mA/cm^2^ for a “simple” columnar thin film (4.3 μm) to 10.6 mA/cm^2^ when adding the NPs (4.3 μm). The differences in terms of light absorption appear to be the main explanation for the different *J*_sc_ values that are measured, whereas for the screen-printed NPs film (9.6 µm), which absorbs the light one order of magnitude better than the SCM photo-anode, the *J*_sc_ value and *η* are only improved by a factor of 4. These results suggest that for the screen-printed NPs, in spite of the amount of dye adsorbed, which is much larger, many of the generated charge carriers are not collected, which is in line with the structural disorder of NPs that contributes to the loss of charges.

These results can be explained by the synergistic effect between the monocrystalline column, which act as extremely good conductive media for the electron, while the additional nanoparticles in the inter-columnar voids help to graft more dye and, therefore, to increase the amount of generated photoelectrons. On the contrary, for a conventional nanoparticle anode, the adsorption of the dye is of very good quality, but the generated electrons are lost due to the already mentioned defective structure of the anode. These mechanisms are depicted in Figure 21b–d. It is worth stressing that the comparison between the different photoanode architectures was not made with films of the same thickness, owing to the following assumption: It is accepted that *J*_sc_ linearly increases as a function of the thickness of the nanoparticle-based thin film, before reaching a plateau and finally decreasing [69]. As the thicknesses considered in our work (<10 µm) still belong to the linear region, the recombination rate is not yet a limiting parameter, allowing our discussions and conclusions to be meaningful.

We can, therefore, conclude that, in this case of study, the integration of NPs allows an increase in the light absorption by improving the dye impregnation, while the nano-sculpted thin film allows an efficient collection and transfer of charges, avoiding the recombination reactions. Generating this synergistic effect between the nanoparticles and the single crystalline columns seems to be a good strategy to ultimately increase the overall efficiency of DSSCs.

## 4. Conclusions

This work summarizes our recent research related to the development of nano-sculpted thin films by magnetron-sputtering-related technologies and to their use in energy-related applications. We first describe our understanding of the growth mechanism associated with the novel utilization of the glancing-angle geometry in magnetron sputtering processes, the MS-GLAD process. The synthesis of model nano-sculpted Ti and Mg films was investigated by using a joint experimental–modeling approach based on kMC simulations implemented in the NASCAM code.

Based on the different morphological properties of Ti and Mg coatings grown for similar experimental conditions, it appears that the homologous temperature as defined in structural zone models, *T*_s_/*T*_m_, is one of the key parameters to finely control the growth of nano-sculpted coatings by MS-GLAD. When comparing the two considered metals, this parameter is different enough to allow for different growth regimes, from Zone 1 for Ti to Zone 2 for Mg. Basically, this parameter mainly defines the importance of the adatom diffusion processes that is, at room temperature, negligible in the Ti case (the ballistic deposition approximation is sufficient), while events such as hops up and hops down from one atomic layer to another should be taken into account to accurately describe the growth of Mg nano-sculpted thin films. This rationalization based on the homologous temperature of the deposited material can even been extended when considering a different chemistry of the system, i.e., an oxidation of the deposited material. Indeed, in such a situation, it is shown that, as expected by the values of the homologous temperature, Ti and TiO_2_ behave almost similarly while a strong impact is observed for Mg.

The other key parameters are related to the collimated nature of the depositing flux and on its impact on the shadowing effect, which is the basic effect when GLAD geometry is considered. Therefore, it has been demonstrated that the deposition pressure, which strongly affects the collimated character of the depositing flux through the mean free path of the particles, has to be low enough (<0.26 Pa) to trigger the formation of the different nano-sculpted structures.

In comparison with the conventional combination of GLAD with evaporation, it is shown that the utilization of magnetron sputtering in given conditions allows for a good crystallization of the deposition material, which is important in many applications. In particular, for TiO_2_ thin films, we demonstrate that anatase monocrystalline-like nanocolumns-based thin films can be synthesized.

From an application point of view, nano-sculpted TiO_2_ coatings were integrated into the photo-anode of dye-sensitized solar cells (DSSCs). First, it appears that the devices based on nano-sculpted thin films outperform nanoparticles-based DSSCs both in terms of charge harvesting and charge recombination. However, the photo-anode thickness drastically affects the cell performances, indicating that the critical parameter is the adsorbed dye density. This problem has been addressed by combing the nano-sculpted TiO_2_ films with a spin-coated TiO_2_ nanoparticles solution. This hybrid system demonstrates a synergetic effect between the columnar thin film and the absorbed nanoparticles, which significantly improved the efficiency of the DSSCs by simultaneously enhancing the charge transport and the quantity of adsorbed dye molecules.

We believe that the development of novel magnetron sputtering approaches to design materials presenting a well-defined morphology at the nanoscale consists of an important opportunity for this well-established technology.

## Supplementary Materials

The following are available online at https://www.mdpi.com/2079-4991/10/10/2039/s1, Figures S1 and S2: Cross-sectional SEM images with the corresponding simulations of Ti and Mg thin films synthesized at various angles of deposition; Figure S3: Angular distribution of particles calculated by SIMTRA for various angles of deposition; Figure S4: Cross-sectional SEM images with the corresponding simulations of Mg thin films synthesized at various deposition pressures.
