# Supplementary material for: Recent Advances in the Development of Nano-Sculpted Films by Magnetron Sputtering for Energy-Related Applications
Source: Nanomaterials (Basel). 2020 Oct 15;10(10):2039. doi: 10.3390/nano10102039 (PMC7602882; doi:10.3390/nano10102039)
Supplement: Supplementary file 1 [file nanomaterials-10-02039-s001.pdf]

nanomaterials

MDPI

Supplementary Materials

Recent Advances in the Development of Nano-Sculpted Films by Magnetron Sputtering for Energy-Related Applications

Adriano Panepinto [1,*] and Rony Snyders [1,2]

[1] Chemistry of Plasma-Surface Interactions, University of Mons, 23 Place du Parc, 7000 Mons, Belgium; rony.snyders@umons.ac.be

[2] Materia Nova Research Center, Chemistry of Plasma-Surface Interactions, 3 Avenue Nicolas Copernic, 7000 Mons, Belgium

* Correspondence: adriano.panepinto@umons.ac.be; Tel.: +32-(65)-554945

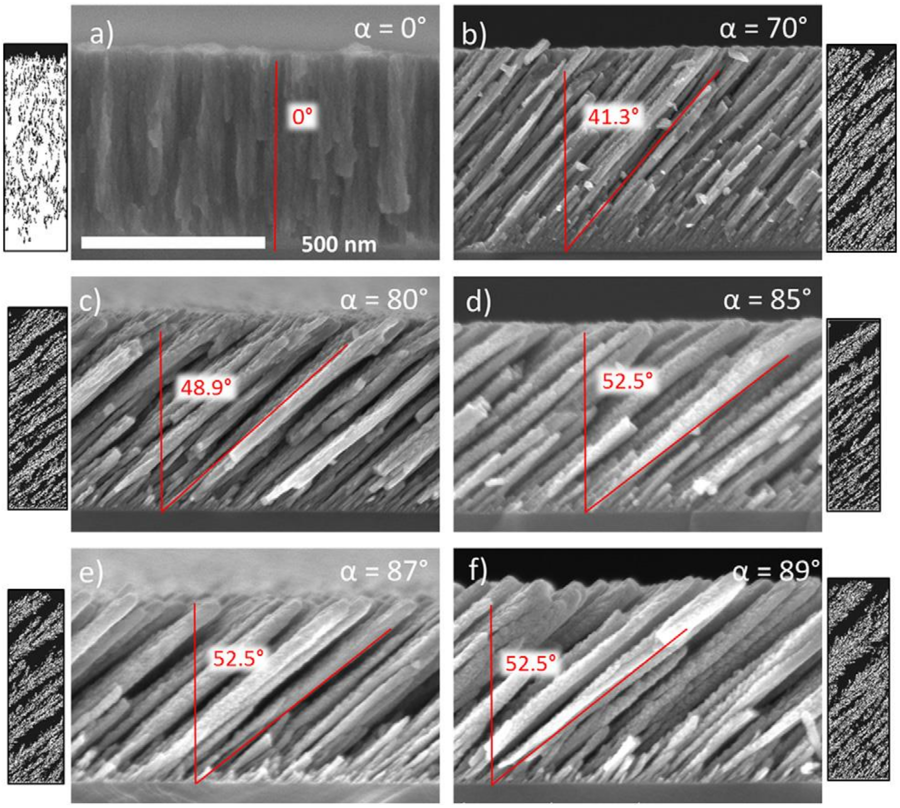

Figure S1. Ti thin films synthesized at 150 W, 0.13 Pa for various angle of deposition (α): (**a**) 0°; (**b**) 70°; (**c**) 80°; (**d**) 85°; (**e**) 87°; (**f**) 89° with the corresponding simulations. The red angle accounts for the average columnar tilt angle (β) estimated by over 20 columns.

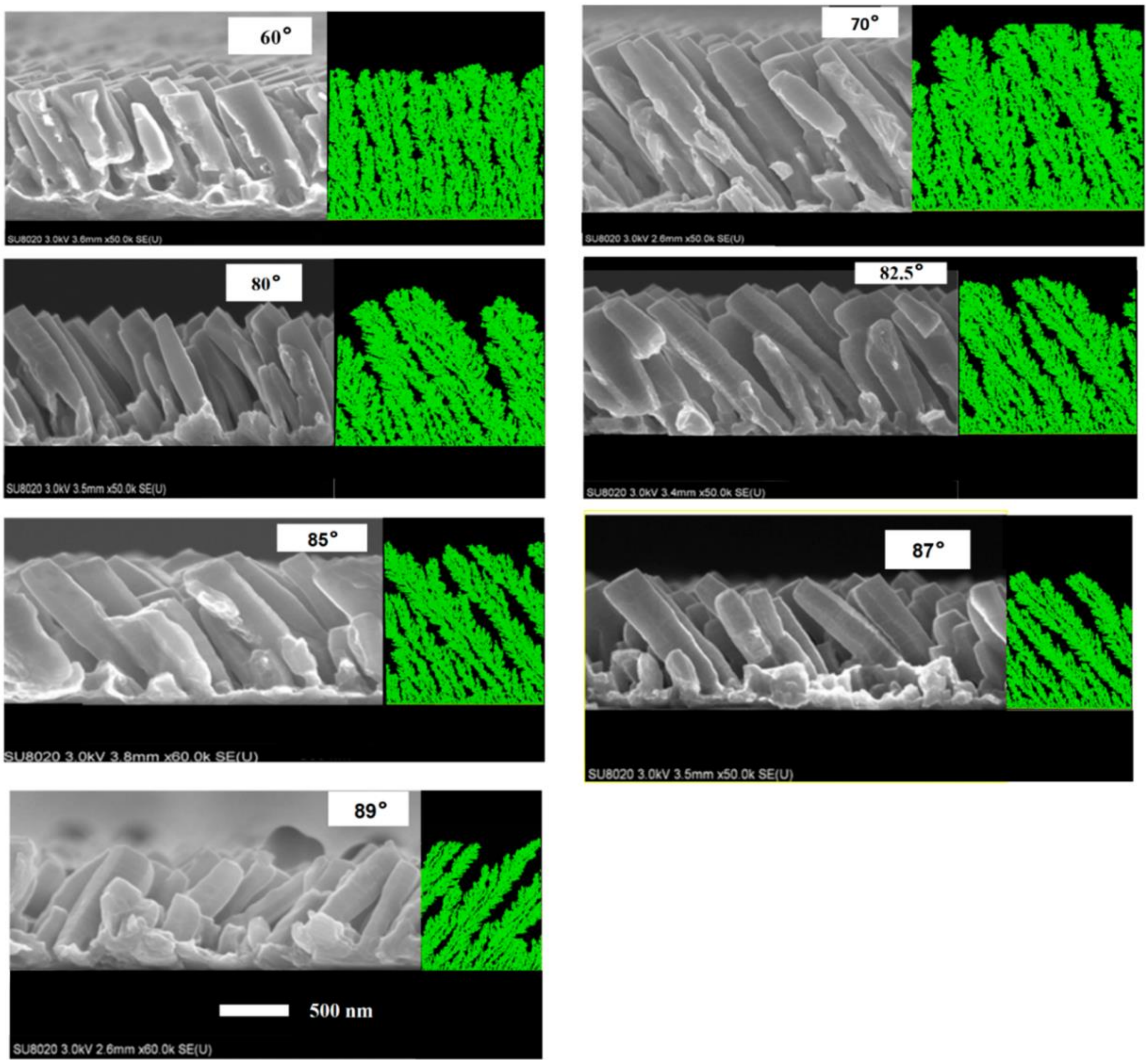

Figure S2. Mg thin films synthesized at 50 W, 0.26 Pa for various angle of deposition (from 60° to 89°) with the corresponding simulations.

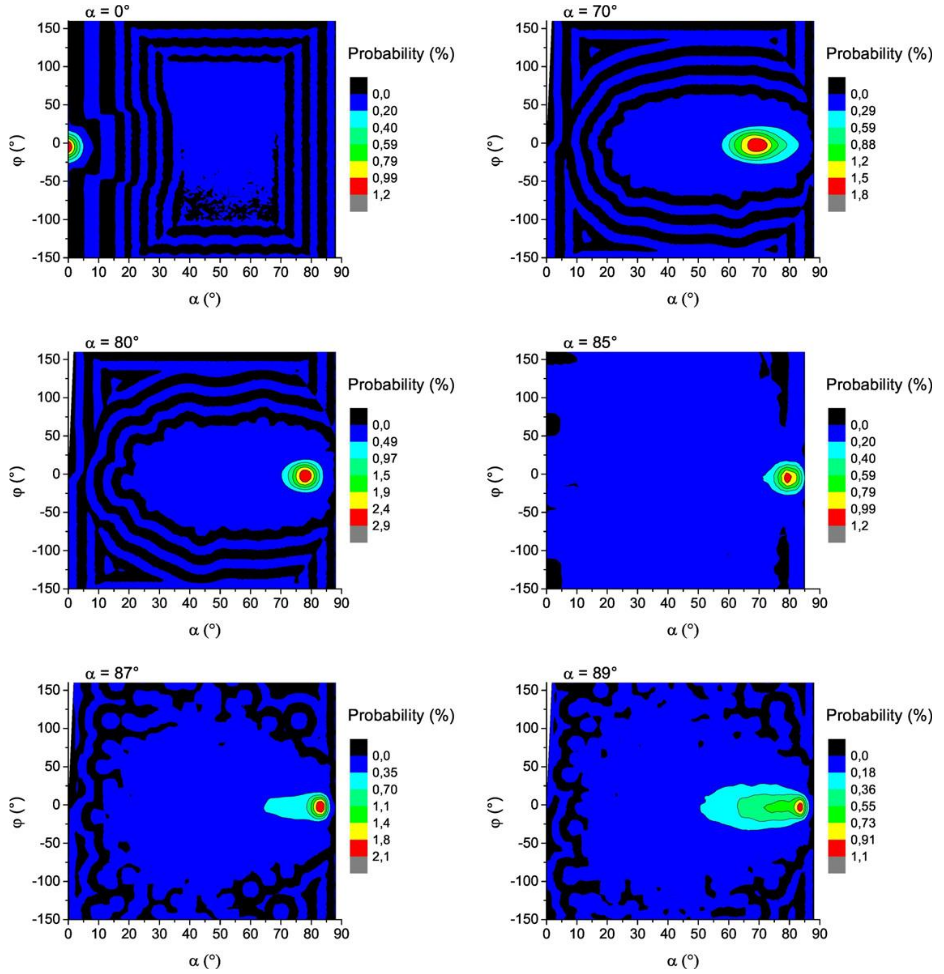

Figure S3. Angle distribution of particles reaching the substrate deposited at 0.13 Pa and 150 W calculated by SIMTRA (simulation of the metal transport) for various angle of deposition α = 0, 70, 80, 85, 87, 89°.

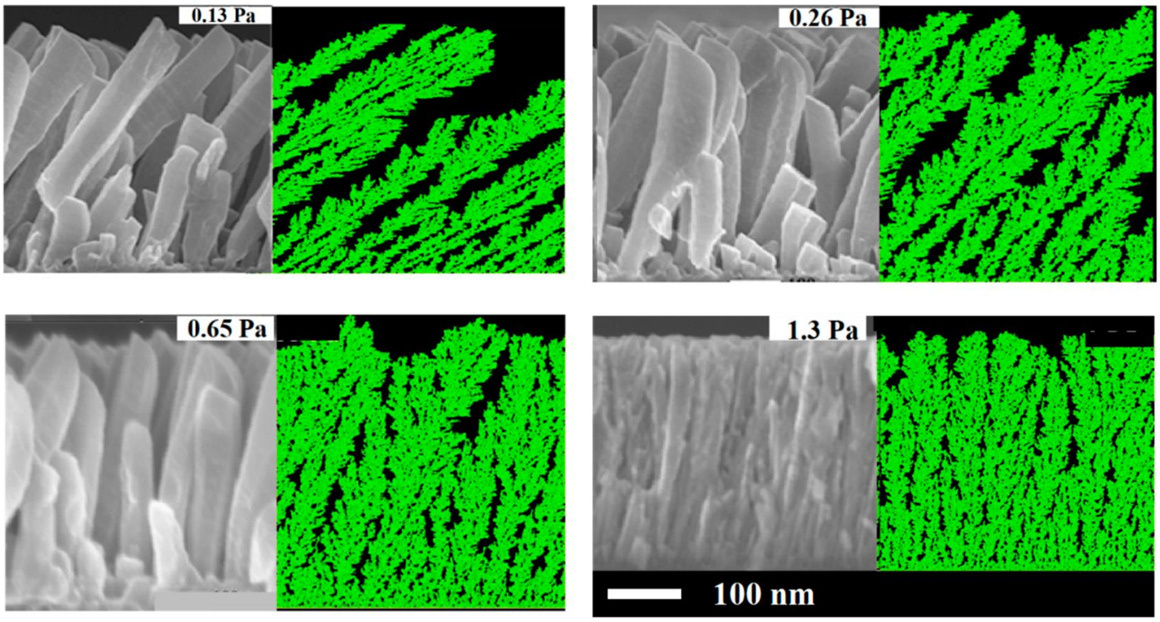

Figure S4. Mg thin films synthesized at 50 W and 85° for various deposition pressure (from 0.13 Pa to 1.30 Pa) with the corresponding simulations.

Publisher's Note: MDPI stays neutral with regard to jurisdictional claims in published maps and institutional affiliations.

© 2020 by the authors. Submitted for possible open access publication under the terms and conditions of the Creative Commons Attribution (CC BY) license (http://creativecommons.org/licenses/by/4.0/).
